# Comparative Analysis of Gut Microbiota between Wild and Captive Golden Snub-Nosed Monkeys

**DOI:** 10.3390/ani13101625

**Published:** 2023-05-12

**Authors:** Yunting Wang, Xuanyi Yang, Mingyi Zhang, Huijuan Pan

**Affiliations:** School of Ecology and Nature Conservation, Beijing Forestry University, Beijing 100083, China

**Keywords:** golden snub-nosed monkey, captive, wild, gut microbiota

## Abstract

**Simple Summary:**

Golden snub-nosed monkey is a critically endangered primate. Determining the gut microbial diversity, construction, and function is vital for protecting the golden snub-nosed monkey. The gut microbiota plays an essential role in regulating the physiological health of wild animals. The dominant phyla in the gut microbiota of captive and wild golden snub-nosed monkeys are *Bacteroidetes*, *Firmicutes*, and *Kiritimatiellaeota*. There are significant differences in the alpha and beta diversities of the gut microbiota between wild and captive golden snub-nosed monkeys, with the captive population having higher alpha diversity than the wild population. Functional predictions related to the Kyoto Encyclopedia of Genes and Genomes (KEGG) database showed that the most significant functional pathway at the second level between captive and wild monkeys was carbohydrate metabolism.

**Abstract:**

Environmental shifts and dietary habits could directly affect the gut microbiota of animals. In this study, we investigated the gut microbiota of golden snub-nosed monkeys under two different conditions: captive and wild. Our study adopted a non-invasive sampling method, using full-length 16S rRNA Pacbio SMAT sequencing technology to compare the gut microbiota of wild and captive golden snub-nosed monkeys. The results showed that the captive populations had higher alpha diversity than the wild populations, and there were also significant differences in beta diversity. The linear discriminant analysis effect size (LEfSe) analysis showed 39 distinctly different taxonomic units. At the phylum level, the most dominant bacteria under captive and wild conditions were Bacteroidetes and Firmicutes. This study revealed that the different fiber intake between wild and captive populations might be the main reason for the difference in the gut microbiota. We found that captive golden snub-nosed monkeys had less beneficial bacteria and more potentially pathogenic bacteria than wild ones. Functional predictions showed that the most significant functional pathway at the second level between the captive and wild monkeys was carbohydrate metabolism. Therefore, our results indicate that diet changes caused by captivity could be the main reason impacting the gut microbiota of captive golden snub-nosed monkeys. We further highlight the potential impact of diet changes on the health of captive golden snub-nosed monkeys and offer some suggestions for the feeding of captive golden snub-nosed monkeys.

## 1. Introduction

Golden snub-nosed monkey (*Rhinopithecus roxellana*) is one of the most endangered species in China [1]. It is only distributed in a remote mountainous area at elevations between 1500 and 3500 m in Sichuan, Gansu, Shaanxi, and Hubei provinces, with approximately 22,000–23,000 individuals in the wild [2]. They usually occupy large home ranges, live in family units, and have complex social relationships [3,4]. As an iconic endangered species and flagship protected species in China, the government has adopted both in situ and ex situ conservation strategies of the golden snub-nosed monkey. Ex situ conservation has been widely applied in protecting endangered animals that have difficulty surviving and reproducing in the wild [5,6]. However, living in a human-controlled environment under ex situ conservation could cause problems in animal health, such as gut microbial disorder, which currently causes significant concerns in wildlife conservation [7,8,9].

The gut microbiome is composed of bacteria, archaea, viruses, and eukaryotic microbes, and they have tremendous potential to impact our physiology, both in healthy and disease conditions [10,11]. The gut microbiota and the host interact through a long-term coevolutionary process to form a complex and relatively stable microbial environment [12]. The gut microbiota plays a vital role in the maintenance of the normal life activities of the host. It has been demonstrated that the gut microbiota is essential in the host’s ecological adaptation, such as immunity digestion, diet behavior, and metabolism [13,14,15,16,17,18,19,20,21].

Significant differences exist between wild and captive lifestyles. The latter includes contact with humans, antibiotic exposure, altered diet composition, and increased stress, which may lead to microbiome disruption in captive animals [22]. Multiple studies have proved that captivity leads to diet alternations, and the living environment can significantly affect the animal’s gut microbiota. Some studies on mammals demonstrated significant differences in the relative abundance of Firmicutes and Bacteroidetes between captive and wild individuals [9,23]. However, the influence of the external environment on the gut microbiota is not limited to the changes in the abundance of specific species or genera. For example, a previous study on wild and captive sika deer (*Cervus nippon*) has proved that captivity could also affect the diversity of the gut microbiota [24]. In studies of non-human primates (NHPs), captivity altered the original structure of the gut microbiome shaped by natural dietary sources [9,25,26,27] and increased the abundance and diversity of antibiotic genes [28]. Previous studies on humans and animals have suggested that many gastrointestinal (GI) diseases and metabolic diseases are strongly connected with gut microbiome disruption [29,30,31,32]. In brief, captivity affects the host’s health by disrupting the gut microbiome. Such disruption causes alterations in the gut microbiota and can lead to pathogen colonization [33,34,35,36], which makes captive animals more susceptible to disease. In addition, the gut microbiota can also significantly affect the metabolism of the host [37]. The metabolite production of the gut microbiota from dietary sources will ultimately affect host health [38]. As a typical folivorous primate, the main food for golden snub-nosed monkeys in the wild environment is a large number of leaves [2], while in captivity, they have less intake of cellulose and protein and more intake of carbohydrates and fat [39,40]. Close contact with humans has increased the probability of infection with pathogenic bacteria. All of these factors will lead to changes in the intestinal tract microorganisms and affect health [17].

Therefore, studying the gut microbiota of golden snub-nosed monkeys in the wild and captivity is essential for protecting this species. In our study, we adopted a non-invasive sampling method that is harmless to animals, using full-length 16S rRNA Pacbio SMAT sequencing technology to compare the gut microbiota of 19 healthy wild and captive golden snub-nosed monkeys. This can help us better understand the adverse effects of captivity on the health status of golden snub-nosed monkeys and provide some feasible suggestions for managing captive golden snub-nosed monkeys.

## 2. Materials and Methods

### 2.1. Samples Collection

A total of 19 fecal samples were collected from 9 wild and 10 captive healthy golden snub-nosed monkeys. The captive samples were collected from Shanghai Wild Animal Park; the wild samples were collected from Mianyang, Sichuan Province. All fecal samples were collected and preserved in 15 mL centrifugal tubes immediately after defecation, snap-frozen in liquid nitrogen, and stored at −80 °C. DNA samples were stored frozen (−20 °C) until use.

### 2.2. DNA Extraction 

Following the protocols provided by the manufacturer, the QIAamp DNA Stool Mini Kit (Qiagen, Valencia, CA, USA) was utilized to extract DNA. The concentration of DNA was assessed utilizing the Qubit dsDNA HS Assay Kit and Qubit 3.0 Fluorometer (Invitrogen, Thermo Fisher Scientific, Waltham, MA, USA). To determine the quality and quantity of the DNA, both a Nanodrop (ND-1000) spectrophotometer (Nanodrop Technologies, Wilmington, DE, USA) and agarose gel electrophoresis methods were utilized.

### 2.3. 16S rRNA Genes Amplicon Sequencing and Bioinformatics Analysis

The 16S rRNA gene was amplified using barcoded 27 forward and 1492 reverse primers (27F:5′-AGAGTTTGATCCTGGCTCAG-3′; 1492R:5′-CTACGGCTACCTTGTTACGA-3′) and sequenced using PacBio Sequel. 

PCR amplification was carried out by performing 25 cycles using a KOD One PCR Master Mix (from TOYOBOLife Science, Shanghai, China). Initial denaturation was at 95 °C for 2 min, followed by denaturation at 98 °C for 10 s, annealing at 55 °C for 30 s, extension at 72 °C for 1 min and 30 s, and a final extension at 72 °C for 10 min. Following purification with Agencourt AMPure XP Beads (supplied by Beckman Coulter, Indianapolis, IN, USA), the total number of PCR amplicons was measured using the Qubit dsDNA HS Assay Kit and Qubit 3.0 Fluorometer (manufactured by Invitrogen, Thermo Fisher Scientific, Waltham, MA, USA). Following individual quantification, the amplicons were combined in equal quantities, and then SMRTbell libraries were generated from the amplified DNA using the SMRTbell Express Template Prep Kit 2.0 (from Pacific Biosciences, Menlo Park, CA, USA), as per the manufacturer’s guidelines. After purification, the SMRTbell libraries from the pooled and barcoded samples were sequenced on a PacBio Sequel II 8M cell utilizing the Sequel II Sequencing Kit 2.0 (from Pacific Bioscience, Menlo Park, CA, USA).

Sequencing data were processed using SMRTlink software (version 8.0) to obtain circular consensus sequencing (CCS) reads through the filtering and demultiplexing of raw reads. To increase the reliability of CCS reads, which were generated following the demultiplexing of raw reads using SMRTlink software (version 8.0), we performed quality filtering in Cutadapt (version 2.7) to identify and discard CCS reads that did not match the forward and reverse primers and those falling outside the specified length range of 1200–1650 bp.

Microbiome bioinformatic analysis was performed with QIIME2 v.2020.11 [41]. Sequences were then quality-filtered, denoised, and merged, and chimeras were removed; then, amplicon sequence variants (ASVs) were output using DADA 2 v.1.8 [42,43], and the ASVs with abundance <0.001% were filtered. To generate the taxonomic table, we aligned ASV sequences against the SILVA reference database (version 132) pretrained at 99% sequence identity using the feature-classifier plugin available in QIIME2 [44].

### 2.4. Statistical Analysis

We evaluated the alpha diversity of the microbial communities using Chao1, Shannon, Simpson, Faith’s, and Pielou’s evenness indices, calculated with QIIME2. Additionally, we performed Kruskal–Wallis tests to examine differences in alpha diversity between the two groups [45,46,47,48,49]. We assessed the similarity of community structure among different groups by conducting principal coordinate analysis (PCoA) for which we utilized a Bray–Curtis dissimilarity matrix to estimate beta diversity. Then, we used the pair group method with arithmetic means (UPGMAs) and non-metric multidimensional scaling (NMDS) to analyze the beta diversity [50,51,52]. The ANOSIM (analysis of similarities) was used to evaluate the similarity between different individuals [53]. We graphically represented the relationship between captive and wild samples using a hierarchical clustering method. The samples were clustered using the average method, based on the distance matrix.

Kruskal–Wallis tests were used to detect the abundant differential features between captive and wild groups. LEfSe analysis (https://huttenhower.sph.harvard.edu/galaxy/ (accessed on 15 July 2022)) was performed to reveal the different taxa identified between captive and wild groups. A size-effect threshold of 3.0 on the logarithmic LDA score was used to discriminate functional biomarkers [54]. Student’s *t*-test was used to determine the microbial communities and functional compositions that were significantly different between the two groups (*p*-value < 0.05). To control for multiple comparisons, we performed false discovery rate correction for the obtained *p*-values [55].

PICRUSt2 was used to predict the functional profiles of microbial communities [56]. The functional profiles of the identified taxa were predicted using the KEGG database. The Kruskal–Wallis test was used to calculate the differences in the gut microbiota between the captive and wild monkeys (*p*-value < 0.05).

## 3. Results

### 3.1. Microbial Community Profiles

A total of 174,012 clean reads were obtained from the 16S rRNA of the 19 snub-nosed monkeys (10 captive; 9 wild) (Table S1). We identified 1012 unique ASVs from the 19 fecal samples based on taxonomic annotation, distributed across 14 phyla, 21 classes, 30 orders, 54 families, and 142 genera (Table S2). The results indicated that the detection of bacteria in the samples was comprehensive, with a Good’s coverage of nearly 99% (means ± SD = 98.5 ± 1.80%). The rarefaction curves in Figure S1 indicate that, as sequencing depth increased, the number of observed species also increased; however, the curve eventually plateaued, indicating that the sequencing depth met the requirement for subsequent analysis.

**Figure 1:**
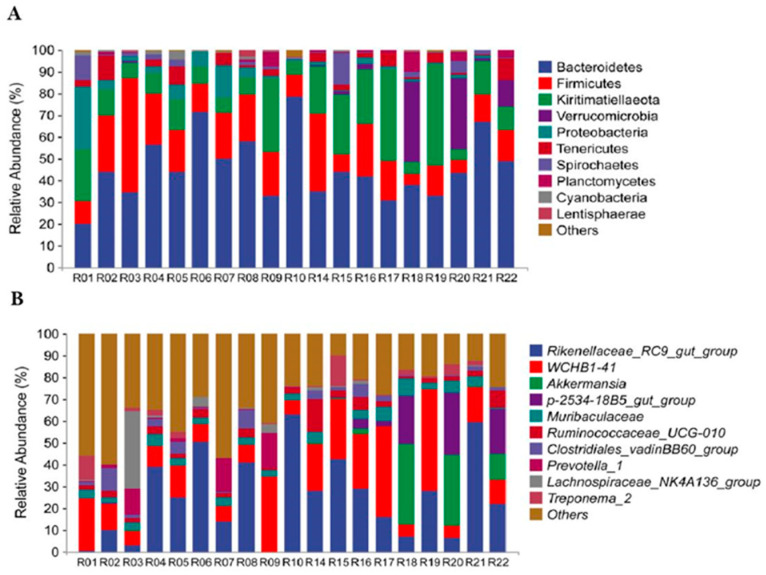
The basic structure of the bacterial community composition of each sample at the phylum (**A**) and genus (**B**) level. Stacked bar graphs illustrate the abundances and the x-axis represents the sample names.

**Figure 2:**
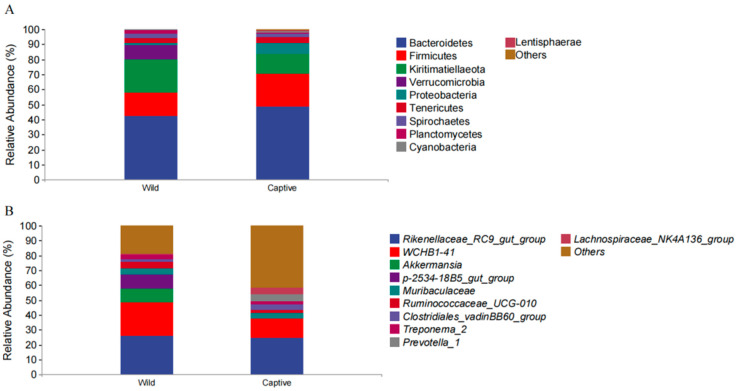
The bacterial community distributions and relative abundances comparison of the two groups at the phylum (**A**) and genus (**B**) level.

The analysis result showed that the top three dominant phyla in both captive and wild groups were *Bacteroidetes* (captive 48.89% vs. wild 42.47%; 45.68% on average); *Firmicutes* (captive 21.74% vs. wild 15.23%; 18.49% on average); and *Kiritimatiellaeota* (captive 13.05% vs. wild 22.21%; 17.63% on average) (Figure 1A,B). At the phylum level, *Bacteroidetes*, *Firmicutes*, *Proteobacteria*, *Tenericutes*, *Cyanobacteria*, and *Lentisphaerae* had higher relative abundance in the captive group, while *Kiritimatiellaeota*, *Verrucomicrobia*, *Spirochaetes*, and *Planctomycetes* had higher relative abundance in the wild group (Figure 2A). At the genus level, the relative abundances of *Clostridiales_vadinBB60_group* and *Prevotella_1* in the captive group were significantly higher than those in the wild group (*p* < 0.05). The relative abundances of *Rikenellaceae_RC9_gut_group*, *WCHB1-41*, *Akkermansia*, *p-2534-18B5_gut_group*, *Muribaculaceae*, *Ruminococcaceae_UCG-010*, *Treponema_2*, and *Lachnospiraceae_NK4A136_group* in the wild group were significantly higher than those in the captive group (*p* < 0.05) (Figure 2B).

### 3.2. Diversity Analysis of Microbiota in Captive and Wild Golden Snub-Nosed Monkey

**Figure 3:**
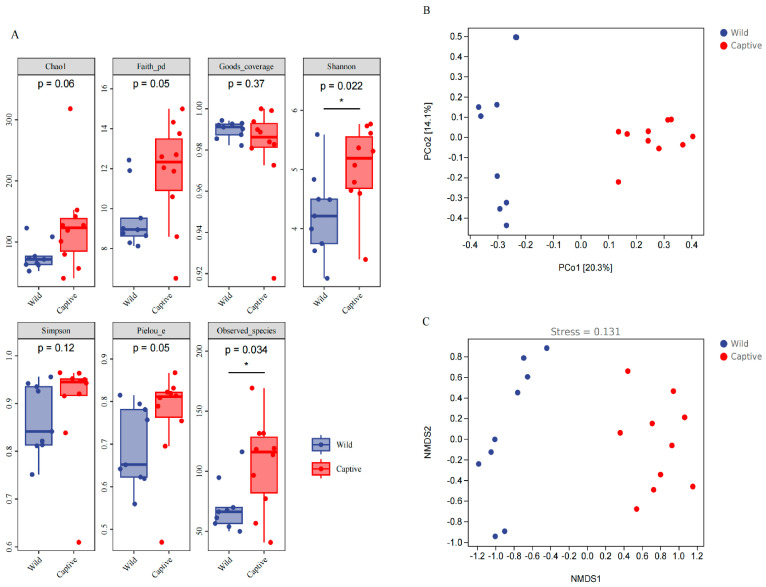
Boxplots showing *α*-diversity indices of microbiota in the captive and wild groups. The indices of Chao 1, Simpson, Shannon, Faith’s PD, Pielou’s evenness, and Good’s coverage. The Chao 1 index is commonly used in ecology to estimate the total number of species; larger Chao 1 values represent the total number of species. The Shannon index and Simpson’s diversity index are standard diversity measures, reflecting the samples’ richness and evenness. * *p* < 0.05 (**A**). The principal coordinate analysis (PCoA) plot of Bray−Curtis distances shows the ecological distance between gut microbial communities in captive and wild monkeys. Blue dots represent wild monkeys, and red dots represent captive monkeys. A closer distance between two points infers a higher similarity (**B**). Using the Bray−Curtis distance, the NMDS plot displays the calculated distance between two groups based on dissimilarity in ASV composition. Blue dots represent wild monkeys, and red dots represent captive monkeys (**C**).

We compared the alpha diversity of the microbiota between the captive group and the wild group based on the abundance at the genus level across all cohorts. The results showed that the captive environment greatly changed the gut microbiome’s alpha diversity of snub-nosed monkeys. We observed a significant difference in the Shannon index (accounts for species richness and evenness) (*p* = 0.022) and the observed species index (accounts for the number of species contained in a community) (*p* = 0.034) between captive and wild monkeys (Figure 3A), indicating high richness, evenness and species number of the microbiota in captive monkeys.

PCoA and NMDS based on Bray–Curtis showed distinct differences in the diversity of the gut microbiota between captive and wild monkeys (Figure 3B,C). The result indicated that the microbial communities from captive monkeys clustered together and were separated from wild monkeys along the principal coordinate axis, which suggests significant differences in the microbial community composition between captive and wild monkeys.

### 3.3. Microbial Taxa Differences in the Gut Microbiota between Wild and Captive Golden Snub-Nosed Monkeys

**Figure 4:**
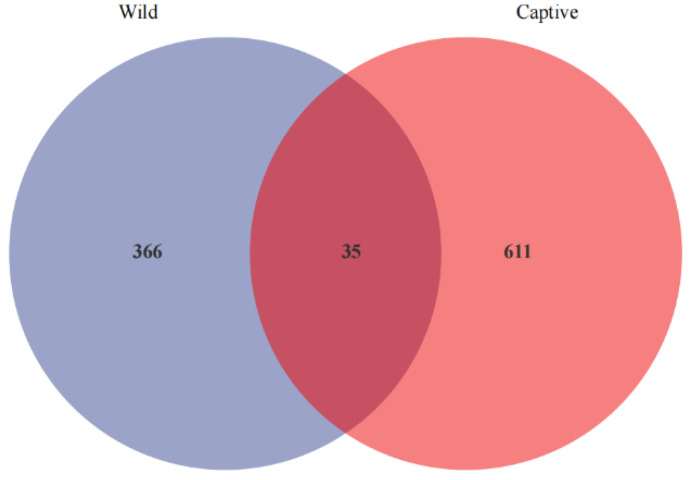
Venn diagram illustrating the number of ASVs shared by the captive and wild groups. The shared taxa by all individuals were to represent the core microbiota in two groups. The 2 groups shared 35 common ASVs, and within each group, there were 611 ASVs in the captive and 366 in the wild group.

The Venn diagram demonstrates the differences in gut microbiota composition between the two groups. A total of 1012 ASVs were detected in the captive and wild monkeys. The captive and wild groups had 35 ASV overlaps, and the captive group had 611 unique ASVs, whereas the wild group had 366 unique ASVs (Figure 4).

**Figure 5:**
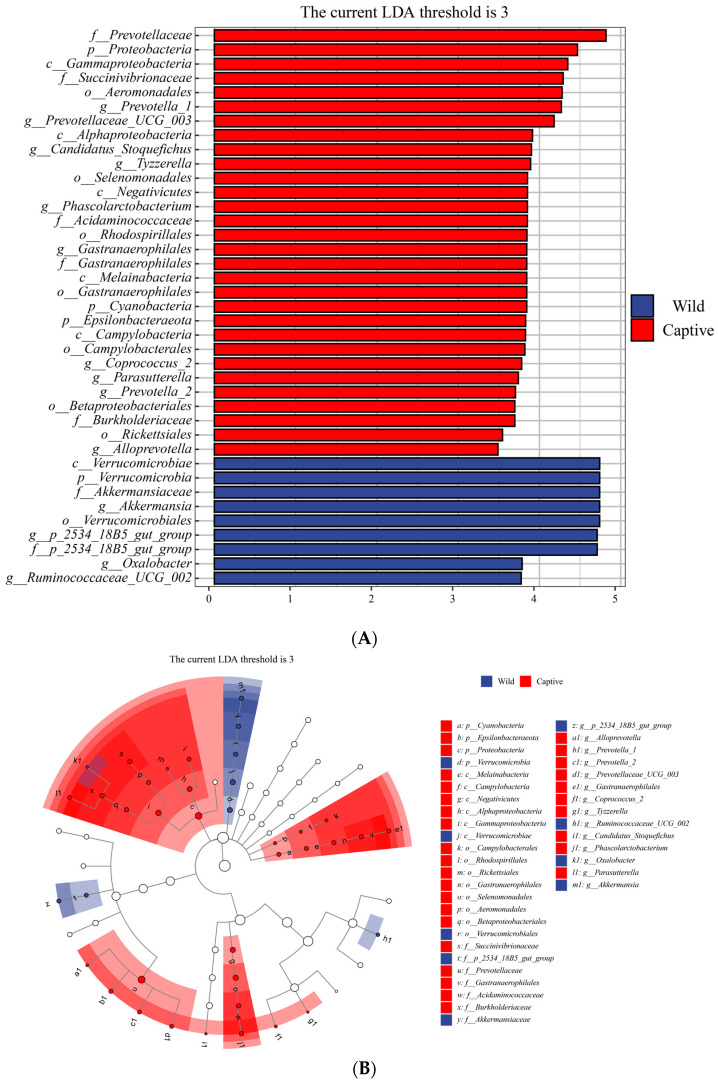
Using the LEfSe analysis to identify the bacterial taxa with significant differences between the two groups. The cladogram shows the evolutionary clades of different species. The circles radiating from the inside to the outside represent the taxonomic level from phylum to genus. Each small circle at a different taxonomic level represents a taxonomy at that level, and the diameter of the circles is proportional to the relative abundance. Red nodes represent microbial groups that play an important role in the captive group, blue nodes represent microbial groups that play an important role in the wild group, and yellow nodes mean no significant difference (**A**). Bacterial taxa with a significant difference and an LDA score greater than the estimated value (3.0); the histogram length represents the LDA score (**B**).

To investigate the potential differences in microbial community composition between captive and wild populations, we utilized LEfSe tests to detect variations in the relative abundance of bacterial taxa. Figure 5 displays the microbial communities with significant differences in the relative abundances between the captive and wild groups. At the phylum level, the relative abundances of *Proteobacteria*, *Cyanobacteria*, and *Epsilonbacteraeota* in the captive golden snub-nosed monkeys were significantly higher than those in wild golden snub-nosed monkeys. In contrast, the relative abundance of *Verrucomicrobia* in wild golden snub-nosed monkeys was significantly higher than that in captive golden snub-nosed monkeys. At the genus levels, the relative abundances of *2534_18B5_gut_group*, *Ruminococcaceae_UCG_002*, *Oxalobacter*, and *Akkermansia* in wild golden snub-nosed monkeys were significantly higher than in captive golden snub-nosed monkeys. At the same time, *Prevotella_1*, *Prevotellaceae_UCG_003*, *Candidatus_Stoquefichus*, *Tyzzerella*, *Phascolarctobacterium*, *Gastranaerophilales*, *Coprococcus_2*, *Parasutterella*, *Prevotella_2*, and *Prevotellaceae_UCG_003* were more abundant in captive golden snub-nosed monkeys.

### 3.4. Functional Differences in Predicted Metagenomic between the Gut Microbiota of Wild and Captive Golden Snub-Nosed Monkeys

**Figure 6:**
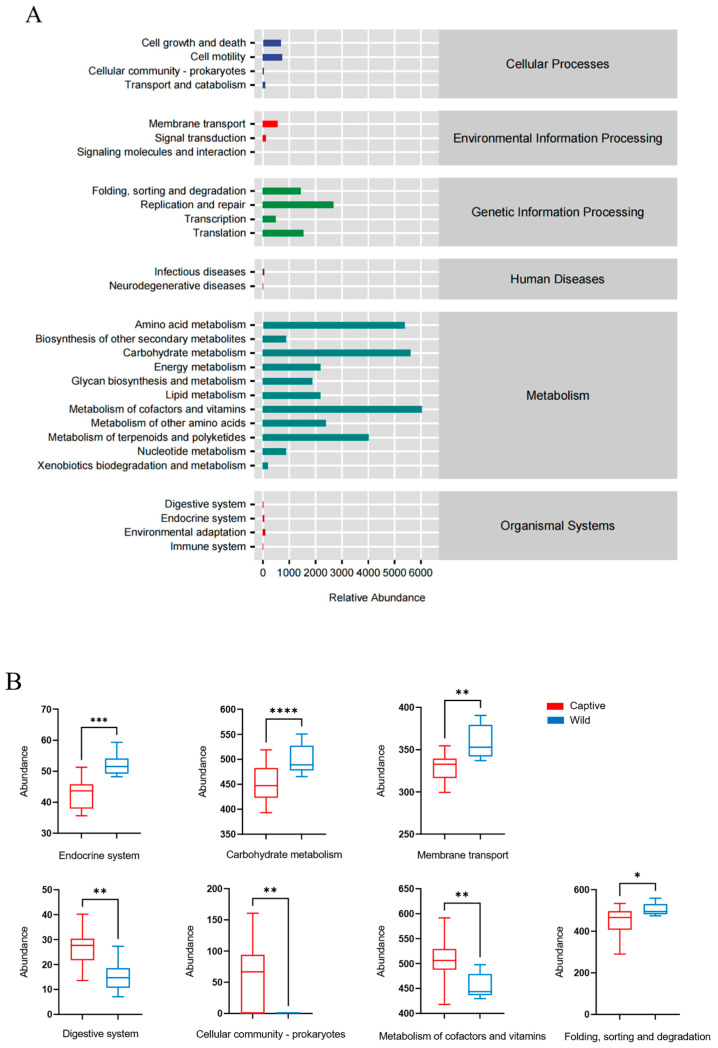
The prediction of the abundance of KEGG pathway classifications for all samples (**A**). Differential functional abundance in RPKM of KEGG Level 2 pathways in the gut microbial between captive and wild monkeys (**B**), * *p* < 0.05, ** *p* < 0.01, *** *p* < 0.001, **** *p* < 0.0001.

We performed a variance analysis of KEGG metabolic pathways in both captive and wild golden snub-nosed monkeys. A total of 129 KEGG Level 3 pathways were annotated, and the Kruskal–Wallis test was utilized to check the significance of K-numbers, where 28 of them were found to be significantly different (*p* < 0.05) between the wild and captive groups (Table S3). The majority of KEGG categories were found to be associated with metabolic processes, genetic information processing, cellular processes, environmental information processing, organismal systems, and human diseases (Figure 6A). The comparative analysis of the second-level pathways showed seven significantly different pathways (*p* < 0.05) (Figure 6B), including carbohydrate metabolism (ko00620), endocrine system (ko04910), membrane transport (ko03070), digestive system (ko04974), cellular community prokaryotes (ko05111), the metabolism of cofactors and vitamins (ko00760), and folding, sorting, and degradation (ko04122) (Table S3). The captive group had a higher abundance in the digestive system, cellular community prokaryotes, and metabolism of cofactors and vitamins. The wild group had a higher abundance of carbohydrate metabolism, endocrine system, and membrane transport (Figure 6B).

## 4. Discussion

In this study, we analyzed the difference in the gut microbiota between wild and captive golden snub-nosed monkeys using full-length 16S rRNA PacBio SMAT sequencing technology. We found that captive life may alter the community structure of the gut microbiota in golden snub-nosed monkeys. In addition, captive golden snub-nosed monkeys have more potential pathogens, which could cause GI problems, indicating that captive life might affect the gastrointestinal health of golden snub-nosed monkeys. Furthermore, our results suggest that diet might be the main cause affecting the gut microbiota of golden snub-nosed monkeys under different circumstances.

In our study, the dominant phyla in both captive and wild golden snub-nosed monkeys were *Bacteroidetes* and *Firmicutes* (Figure 1A), the same as other studies on primate gut microbiota [57,58,59,60,61]. *Bacteroidetes* can help degrade simple sugars, proteins, and carbohydrates, while *Firmicutes* are the main cellulolytic bacteria that can degrade fiber and cellulose [62,63,64,65]. Free-ranging golden snub-nosed monkeys consume a fiber-rich diet in the wild, and their primary food is more diverse, including lichens, leaves, seeds, fruits, buds, and bark [66]. Meanwhile, the diet of captive golden snub-nosed monkeys contains lower crude fiber than natural diets, and captive monkeys only have a mean of 15% crude fiber intake, while that of wild monkeys is up to 52% [39,67]. We found that *Bacteroidetes* were more abundant than *Firmicutes* in captive monkeys (Figure S2), which could be due to captive golden snub-nosed monkeys consuming more carbohydrates than wild monkeys.

We found the taxa with significant differences between the two groups using LEfSe analysis (Figure 5A,B). There are several notable bacterial taxa; some are potential pathogens, some are beneficial for hosts’ health, and some are associated with diet. The genus *Prevotella* was significantly more abundant in captive golden snub-nosed monkeys. The high abundance of *Prevotella* often corresponds to an increased ability to digest simple carbohydrates [68,69,70]. This suggests that in captive individuals, the ability to digest cellulose is reduced as a result of food changes which in turn increases the ability to digest simple carbohydrates. In addition, *Prevotella* has a beneficial impact on glucose metabolism [71,72], and researchers have confirmed the role of *Prevotella* in regulating host health. Therefore, we could speculate that the increased abundance of *Prevotella* in captive monkeys is related to adaptation to captivity. *Proteobacteria* were also observed in the captive group. It is considered to be a marker of microbial dysbiosis and potential diagnostic criteria for disease [73] and is closely correlated with inflammatory bowel disease (IBD) [33,74] and colorectal cancer [75]. A higher *Proteobacteria* ratio is usually connected with poor health conditions [76,77]. *Proteobacteria* also play a role in intestinal inflammation. Studies on immunodeficient mice have pointed to the disturbance of the gut microbiome in diseased immunodeficient mice with a higher proportion of *Proteobacteria* species [78,79]. Another study of Crohn’s disease also showed an increased relative abundance of Proteobacteria [80]. In addition, studies have shown that a high intake of sugars may increase the relative abundance of *Proteobacteria* in the gut [81,82]. Therefore, we can speculate that captive golden snub-nosed monkeys’ high sugar intake (such as fructose and starch) could be the reason for the increased abundance of *Proteobacteria* in the captive group’s gut microbiota. We found that the genus *Akkermansia* was abundant in the wild group. *Akkermansia* is considered a marker of intestinal health [83] and is essential in enhancing glucose tolerance, reducing insulin resistance, and regulating pathways in establishing basal metabolic homeostasis [84]. Studies have confirmed that diet significantly affects *Akkermansia*, and a decreased abundance of *Akkermansia* could be a sign of malnutrition [85].

In the results of our diversity analysis, captive monkeys’ gut microbiota was richer and more diverse than that of wild monkeys. As typical folivorous primates, golden snub-nosed monkeys can obtain a more homogeneous diet in the wild but obtain richer food types in a captive environment; thus, we suggest that this factor may result in a significant increase in their gut microbial species and abundance in their gut [86]. In addition, animals in captivity have more frequent contact with humans and live in a more complex environment. The veterinary treatment of their diseases and the use of drugs may also lead to significant changes in their gut microbiota [87].

Though the findings showed that there was no significant difference in the makeup of functional pathways at the first level of KEGG analysis between the two groups (Figure 6A), gene function predictions showed that the second-level pathway was mainly related to metabolism, suggesting that the gut microbiota are closely related to their natural environments, especially for the host diet [88]. Thus, our study results indicate that gut microbiota plays an essential role in host physiology, and more studies are needed to investigate the mechanism of functional pathways further.

## 5. Conclusions

In summary, our study suggests that the gut microbiota of golden snub-nosed monkeys could be affected by a captive environment, especially due to changes in diet. By comparing the differences in the gut microbiota of both groups, we found a divergence in the diversity of the captive and wild monkeys’ gut microbiota, which could be due to the captive monkeys consuming different food than the wild monkeys. On the one hand, we found that the wild monkeys had unique beneficial bacteria (*Akkermansia*), while the captive monkeys had more potentially pathogenic bacteria, suggesting that captive monkeys have a higher potential to get infected with diseases and suffer from poor health. On the other hand, *Prevotella*, which has a positive effect on glucose metabolism, was found to have a higher proportion in the gut of captive monkeys, indicating adaptation to captivity. The functional prediction analysis further confirmed the functional differences between the microbiota of the captive and wild monkeys. Our study could have implications for the implementation of instructions on how to feed animals in captivity. Overall, we suggest that captivity could disrupt the gut microbiota, but on the other hand, this disruption might help the host adjust to captive life.

## Data Availability

Sequencing data can be found in the Sequence Read Archive (http://www.ncbi.nlm.nih.gov/bioproject/971738) under BioProject PRJNA971738.

## Supplementary Materials

The following supporting information can be downloaded at: https://www.mdpi.com/article/10.3390/ani13101625/s1, Supplementary Figure S1: Rarefaction curves of the observed species index (A) and rank abundance curves of the ASVs (B); Supplementary Figure S2: Bar chart illustrating the relative abundance of Firmicutes and Bacteroidetes in the gut microbiota of captive and wild groups; Supplementary Table S1: Metadata of the samples and data information. Supplementary Table S2: ASVs of each group’s taxonomy. The relative abundance of each taxonomy. Supplementary Table S3: Relative abundance of K-numbers and KO abundance of captive and wild monkeys. KO differs between captive and wild monkeys calculated by the Kruskal–Wallis test. Supplementary Table S4: Numbers of taxonomic units of the samples and alpha diversity index.
