# Supplementary material for: Comparative Analysis of Gut Microbiota between Wild and Captive Golden Snub-Nosed Monkeys
Source: Animals (Basel). 2023 May 12;13(10):1625. doi: 10.3390/ani13101625 (PMC10215246; doi:10.3390/ani13101625)
Supplement: Supplementary file 1 [file animals-13-01625-s001.zip › supplement Figure S1.pdf]

A

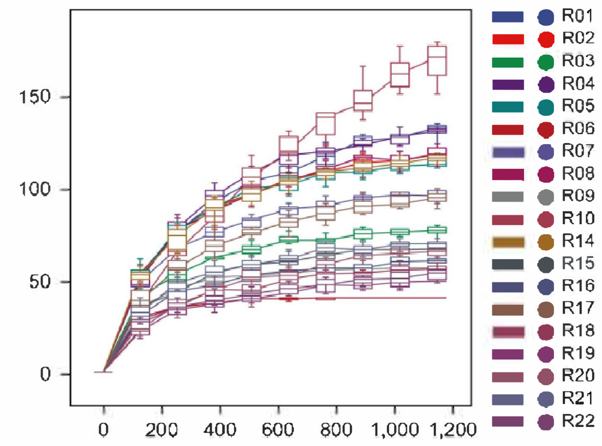

B

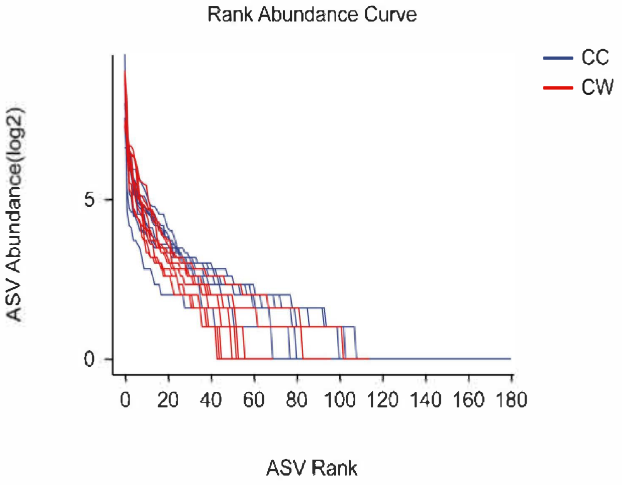

Figure S1. Rarefaction curves of the observed species index. Each curve in the graph represents a sample. The horizontal coordinate shows the number of valid sequences per sample, and the vertical coordinate shows the amplicon sequence variants (ASVs). Different samples are represented by different color curves. The number of ASVs increased as the sequencing depth increased (A).

Rank abundance curves of the ASVs in all 19 samples. The curves reflected the number of high abundance and rare ASVs. The horizontal coordinate shows the ordination number of ASVs and the the vertical coordinate shows the relative abundance. The wideness of the curves reflect the abundance of species, the shape of curves reflect the evenness of species (B).
