# Supplementary figures and images for: Comparative Analysis of Gut Microbiota between Wild and Captive Golden Snub-Nosed Monkeys
Source: Animals (Basel). 2023 May 12;13(10):1625. doi: 10.3390/ani13101625 (PMC10215246; doi:10.3390/ani13101625)

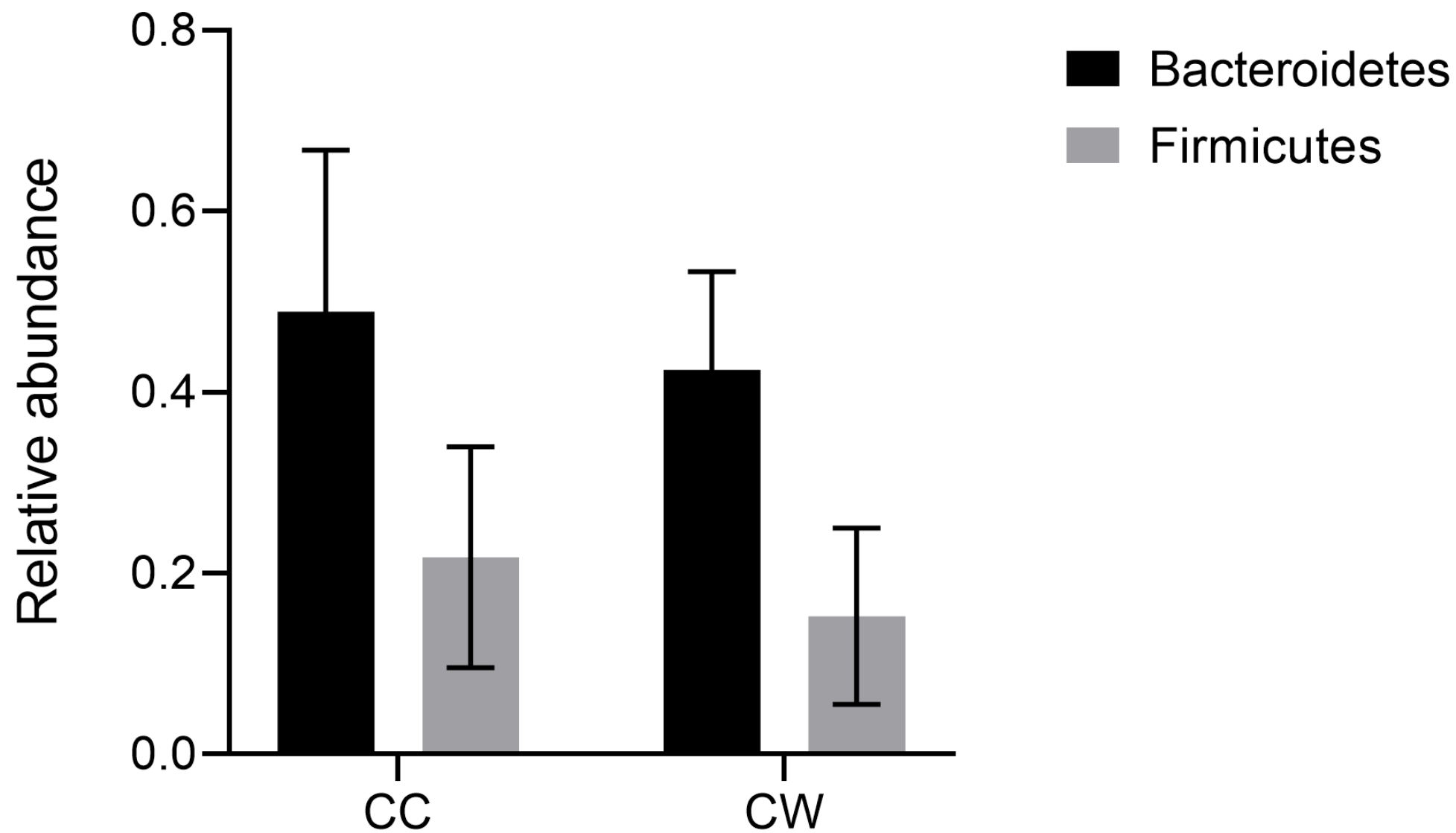

Supplement: Supplementary file 1 [file animals-13-01625-s001.zip › supplement Figure S2.pdf]
